# Global Trends in Incidence of Caries in Permanent Teeth of the Elderly Aged Over 55 Years, 1990–2021: A Multilevel Analysis Using GBD Data

**DOI:** 10.3290/j.ohpd.c_2719

**Published:** 2026-06-19

**Authors:** Siman Sun, Xin Li, Yupei Lai, Anqi Yang, Chu Xu, Zilong Deng

**Affiliations:** a Siman Sun* Dental Researcher, Department of Stomatology, Nanfang Hospital, Southern Medical University, Guangzhou, China; School of Stomatology, Southern Medical University, Guangzhou, China. Statistical analysis, developed the software code and prepared the original draft of the manuscript.; b Xin Li* Dental Researcher, Department of Stomatology, Nanfang Hospital, Southern Medical University, Guangzhou, China; School of Stomatology, Southern Medical University, Guangzhou, China. Data validation, visualisation and interpretation of the results, and assisted in drafting the manuscript.; c Yupei Lai* Dental Researcher, Department of Stomatology, Nanfang Hospital, Southern Medical University, Guangzhou, China; School of Stomatology, Southern Medical University, Guangzhou, China. Data visualisation and critically revised the manuscript for important intellectual content.; d Anqi Yang Dental Researcher, Department of Stomatology, Nanfang Hospital, Southern Medical University, Guangzhou, China; School of Stomatology, Southern Medical University, Guangzhou, China. Data visualisation and critically revised the manuscript for important intellectual content; e Chu Xu Dental Researcher, Department of Stomatology, Nanfang Hospital, Southern Medical University, Guangzhou, China; School of Stomatology, Southern Medical University, Guangzhou, China. Conceptualisation, methodology design and supervision of the study.; f Zilong Deng Dental Researcher, Department of Stomatology, Nanfang Hospital, Southern Medical University, Guangzhou, China; School of Stomatology, Southern Medical University, Guangzhou, China. Study design, data curation and formal analysis. *Siman Sun, Xin Li, and Yupei Lai contributed equally to this work.

**Keywords:** elderly, GBD, global health, incidence, permanent tooth caries

## Abstract

**Purpose:**

To examine global, regional, and national trends in the incidence of permanent tooth caries among adults aged ≥ 55 years from 1990 to 2021 and to evaluate disparities across sociodemographic contexts.

**Methods and Materials:**

Incidence data were obtained from the Global Burden of Diseases 2021 study covering 204 countries and territories. Incidence rates and incident case numbers were analysed at global, regional, and national levels. Temporal trends were assessed using Joinpoint regression to calculate annual percentage change (APC) and average annual percentage change (AAPC).

**Results:**

Globally, the number of caries cases in the elderly increased from 121.54 million in 1990 to 260.18 million in 2021, with a relative change of 114%, while the incidence rate slightly decreased from 18.1% in 1990 to 17.5% in 2021. However, there were differences in trends among different age groups, genders, and regions. The 55–59-year-old group had the highest incident rate. Low-to-middle Socio-demographic Index (SDI) areas had a consistently high incidence. The number of cases increased in all Global Burden of Diseases, Injuries, and Risk Factors Study regions. Joinpoint analysis identified significant temporal change points in multiple regions, indicating divergent epidemiological trajectories.

**Conclusion:**

Permanent tooth caries among adults aged ≥ 55 years continues to represent a significant public health challenge. Although some high-income settings show stabilising trends, increasing incidence in socioeconomically transitioning regions highlights inequalities in preventive coverage and access to care. Targeted, age-specific preventive strategies are essential to address the growing needs of ageing populations.

Population ageing has become one of the most significant demographic transformations worldwide. As life expectancy increases and the proportion of older adults expands, oral health challenges in later life are emerging as a growing public health concern. Dental caries in permanent teeth remains one of the most prevalent chronic conditions globally and disproportionately affects older populations due to cumulative exposure to risk factors, reduced salivary function, comorbidities, and barriers to accessing dental care.^7,13,15^ The prevalence of dental caries rises significantly after the eruption of permanent teeth, peaks in late adolescence and early adulthood, and then continues to increase with age.^28,31^ Unlike many other chronic conditions, untreated caries in older adults can lead to pain, tooth loss, impaired nutrition, and diminished quality of life, thereby contributing to broader health inequalities. Consequently, caries in older populations is no longer merely a residual condition of earlier life but an active and evolving public health challenge.

The Global Burden of Disease (GBD) Study provides a comprehensive framework for evaluating long-term epidemiological trends across countries and regions. Several studies have used GBD data to describe global patterns of oral diseases. However, most existing analyses have either focused on the overall population, broad age categories, or single time points, with limited attention to elderly-specific trends across extended time horizons. In particular, there remains insufficient understanding of how caries incidence among adults aged over 55 years has evolved over the past three decades, especially in the context of rapid demographic ageing and widening socioeconomic disparities between regions.

Furthermore, while previous GBD-based studies have reported overall prevalence or incidence rates, fewer investigations have systematically examined temporal trends using robust statistical modelling approaches capable of identifying significant change points and differential trajectories across sociodemographic strata.^4,34^ Without such analyses, it is difficult to determine whether observed changes reflect gradual shifts, policy-related inflexion points, or persistent structural inequalities.

Addressing these gaps is essential for informing evidence-based oral health policy. As many countries transition from infectious disease-dominated health systems to chronic disease management frameworks, understanding long-term caries patterns among older adults becomes critical for resource allocation, preventive strategies, and integration of oral health into ageing-related public health planning.^8^

To address the aforementioned research gaps, this study is the first to systematically analyse the global trend of caries in the permanent teeth of elderly individuals aged 55 years and above from 1990 to 2021, based on GBD 2021 data.^3^ By stratifying and analysing differences by gender, age group, SDI region, and 21 GBD geographic regions, this study aimed to (1) reveal the spatial and temporal evolution of the caries burden in the elderly; (2) identify high-risk populations; and (3) provide an evidence-based basis for geriatric oral health policies at the global and regional levels.

## METHODS AND MATERIALS

### Study Design and Data Source

This study analysed the global incidence of permanent tooth caries among individuals aged ≥55 years between 1990 and 2021, utilising publicly accessible data sets from the Global Burden of Diseases, Injuries, and Risk Factors Study (GBD). The analysis spanned 204 countries and territories, representing diverse sociodemographic and geographic contexts.^3^

The GBD framework integrates DisMod-MR 2.1 (Institute for Health Metrics and Evaluation, University of Washington), a Bayesian meta-regression platform designed to harmonise epidemiologic data with high sparsity and heterogeneity. This tool employs compartmental differential equation systems and enables robust estimation of incidence rates through the synthesis of input data from disparate sources.^2,3,27^ Uncertainty intervals (UIs) were derived from 1,000 posterior draws, and 95% UIs were defined by the 2.5th and 97.5th percentiles.^3^ The GBD data covered 204 countries and territories, categorised into five Socio-demographic Index (SDI) regions and 21 GBD geographic regions (see Table A4). Detailed country-level and Joinpoint results are provided in the Supplementary Tables (see Tables A1 to A4).

### Case Definition

In GBD, permanent tooth caries is defined as ‘permanent dentition showing unmistakable cavity, undermined enamel, a detectably softened undersides or walls, a tooth with a temporary filling, or a tooth that is filled but also decayed is present’.^32^

### Age Definition

The ≥ 55-year threshold was selected for methodological and epidemiological considerations. The GBD database provides internally modelled incidence estimates for this age category, ensuring consistency with the DisMod-MR framework. In addition, the mid-to-late 50s represent an early ageing transition period characterised by increasing root surface exposure and multimorbidity, which may influence caries susceptibility.^6,11,35^

### Outcome Measures

The primary outcomes were incident case counts and incidence rates per 100,000 population among individuals aged ≥ 55 years.

### Statistical Analysis

Descriptive analyses were conducted to summarise incidence patterns at the global, regional and national levels in 1990 and 2021. Relative percentage changes in incident cases between 1990 and 2021 were calculated to assess long-term variation.

Temporal trends in incidence rates were evaluated using Joinpoint regression analysis (Joinpoint Regression Programme, version 5.3.0.0). This method identifies statistically significant changes in linear trends over time and estimates the annual percentage change (APC) for each segment. The average annual percentage change (AAPC) was calculated to summarise the overall trend across the entire study period. The AAPC was computed as a duration-weighted average of segment-specific APCs, where weights corresponded to the temporal length of individual Joinpoint intervals. Trends were considered statistically significant if the 95% confidence interval of the APC or AAPC did not include zero. In addition, temporal trends were classified based on the APC and its 95% CI: an increasing trend was defined as an APC greater than 0 with a 95% CI that did not include 0, and a decreasing trend as an APC less than 0 with a 95% CI that did not include 0. If the 95% CI included 0, the trend was considered stable. Geographical distribution maps were generated using R software (R Foundation for Statistical Computing).

## RESULTS

### Global Trends

**Fig 1:**
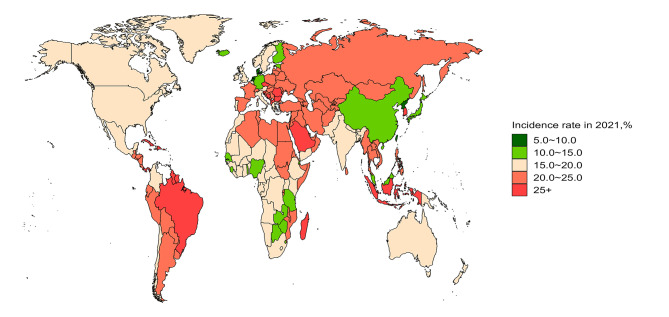
Global map of percentages of relative change in incidence rate of caries in permanent teeth in people aged over 55 years in 2021 in 202 countries and territories.

**Fig 2:**
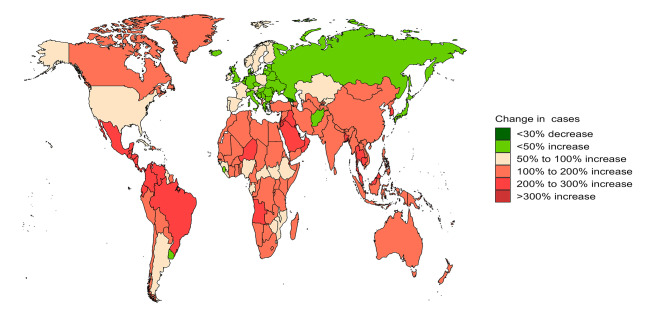
Map of the relative change in cases of incidence numbers of caries in permanent teeth in people aged over 55 years in 202 countries and territories.

**Fig 3:**
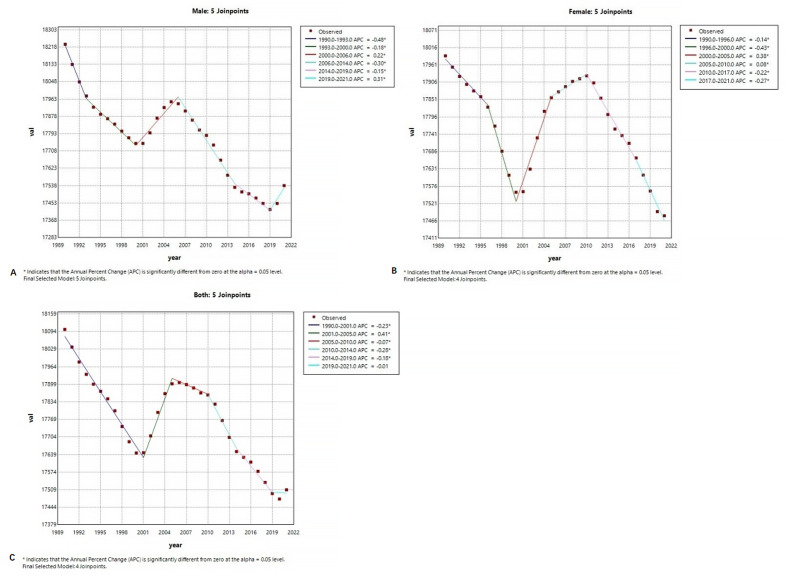
The Annual Percent Change(APC) of incident rates of caries in permanent teeth in people aged over 55 from 1990 to 2021.

The number of cases of dental caries among people aged 55 and above worldwide increased from 121.54 million (95% UI: 99.22 to 150.53) in 1990 to 260.18 million (95% UI: 213.48 to 320.75) in 2021, representing an increase of 114.06% (95% UI: 41.81 to 223.18%) (see Table 1). The incidence rates of dental caries among people aged 55 and above worldwide were 18.0% (95% CI 14.8% to 22.2%) in 1990 and 17.5% (95% CI 14.4% to 21.6%) in 2021, showing a slight overall decrease. However, differences in trends existed among different age groups (see Table 2). The Joinpoint analysis revealed a statistically significant decline (APC = –0.23%, 95% CI –0.24% to –0.21%) of incidence rates during the initial study phase (1990–2001). This downward trajectory was interrupted by a brief increase at an APC of 0.41% (95% CI 0.31% to 0.52%) from 2001 to 2005. Then the APC entered a fluctuating decreasing stage, where the APC was –0.28% (95% CI –0.38% to –0.18%) from 2010 to 2014, –0.18% (95% CI –0.25% to –0.12%) from 2014 to 2019, and nearly stable from 2019 to 2021 (see Table A1 and Figs 1 to 3).

**Table 1:** Number of incident cases worldwide

Characteristics	Incident Cases, (95% Uncertainty Interval)		Relative Change, % (95% Uncertainty Interval)
	1990	2021	
**Overall**	121540805.3 (99219086.2 - 150533948.7)	260176273.9 (213478411.3 - 320753893.4)	114.06 (41.81 - 223.28)
**Sex**			
Female	64752203.1 (53261842.0 - 79836323.2)	137492690.0 (112929684.9 - 169612720.7)	112.34 (41.45 - 218.45)
Male	56788602.2 (46123363.9 - 70995583.1)	122683583.9 (100281115.0 - 151445210.9)	116.04 (41.25 - 228.35)
**Age**			
55 - 59 years	39127120.2 (29843800.3 - 50303133.9)	83070474.3 (63167534.1 - 106518453.7)	112.31 (25.57 - 256.92)
60 - 64 years	30494415.7 (23411067.8 - 39005392.0)	60798347.3 (47115300.0 - 77612426.2)	99.38 (20.79 - 231.52)
65 - 69 years	21412646.7 (17046384.4 - 27611119.3)	46411497.7 (37007764.6 - 59582908.9)	116.75 (34.03 - 249.53)
70 - 74 years	13352878.3 (10241022.4 - 17760877.5)	31469299.8 (24484669.1 - 41314579.6)	135.67 (37.86 - 303.42)
75 - 79 years	9414369.2 (7198791.3 - 12281147.2)	18768013.5 (14579538.1 - 24471217.6)	99.35 (18.71 - 239.94)
80 - 84 years	5098490.2 (3893453.5 - 6552459.0)	11623756.2 (8956044.9 - 14947420.3)	127.98 (36.68 - 283.91)
85 - 89 years	2032043.6 (1549557.6 - 2602858.1)	5570055.7 (4326156.1 - 7075925.4)	174.11 (66.21 - 356.64)
90 - 94 years	508774.2 (364850.0 - 674121.5)	1962618.0 (1413186.6 - 2596897.0)	285.75 (109.63 - 611.77)
95+ years	100067.2 (55211.3 - 153612.8)	502211.4 (282028.7 - 766052.9)	401.87 (83.60 - 1287.49)
**Sociodemographic Index Region**			
Low SDI	7070138.1 (5831923.9 - 8663624.8)	14938424.7 (12336597.9 - 18258289.9)	111.29 (42.40 - 213.07)
Low-to-middle SDI	20634725.1 (16788155.5 - 25846614.9)	48584115.5 (39748453.9 - 60439251.0)	135.45 (53.79 - 260.01)
Middle SDI	29495501.2 (23794431.0 - 36899739.3)	83680817.1 (67416105.9 - 104806489.4)	183.71 (82.70 - 340.47)
High-to-middle SDI	31672072.6 (25765159.5 - 39695335.3)	56665237.6 (46374353.6 - 70405380.1)	78.91 (16.83 - 173.26)
High SDI	32483878.1 (26665903.0 - 40282914.7)	56005170.5 (46512345.0 - 68434610.1)	72.41 (15.46 - 156.64)
**Global Burden of Diseases, Injuries, and Risk Factors Study Region**			
Andean Latin America	785740.1 (615828.6 - 991652.2)	2311000.0 (1801221.7 - 2893154.6)	194.12 (81.64 - 369.80)
Australasia	720095.0 (557476.3 - 952604.5)	1583569.7 (1235272.5 - 2071945.5)	119.91 (29.67 - 271.67)
Caribbean	1175590.0 (966947.0 - 1455243.0)	2357713.4 (1953246.9 - 2855076.4)	100.56 (34.22 - 195.27)
Central Asia	1946291.5 (1551349.9 - 2464627.5)	3564180.6 (2786300.4 - 4581405.5)	83.13 (13.05 - 195.32)
Central Europe	6534386.5 (5326901.9 - 8093148.2)	9005532.4 (7444849.7 - 11164166.0)	37.82 ( - 8.01 - 109.58)
Central Latin America	2820319.8 (2267549.1 - 3581877.0)	8886303.0 (7154476.4 - 11255726.9)	215.08 (99.74 - 396.38)
Central Sub-Saharan Africa	674556.8 (517826.5 - 891758.4)	1622403.9 (1264222.7 - 2172856.5)	140.51 (41.77 - 319.61)
East Asia	15233534.1 (11913801.0 - 19398549.3)	41952194.8 (32770669.8 - 53608281.9)	175.39 (68.93 - 349.97)
Eastern Europe	11849499.8 (9449344.8 - 15262762.2)	14599452.1 (11763649.2 - 18483081.9)	23.21 ( - 22.93 - 95.60)
Eastern Sub-Saharan Africa	2239571.7 (1828890.3 - 2742406.9)	4818413.7 (3983148.5 - 5937767.6)	115.15 (45.24 - 224.67)
High-income Asia Pacific	6072035.7 (4686565.1 - 7781651.2)	9919356.9 (7976435.5 - 12156538.7)	63.36 (2.50 - 159.39)
High-income North America	10007173.0 (7592597.0 - 13177757.8)	19560553.5 (15111033.2 - 25118487.7)	95.47 (14.67 - 230.83)
North Africa and Middle East	6272334.4 (5078360.9 - 7773767.9)	16788163.0 (13696139.0 - 20832815.5)	167.65 (76.18 - 310.23)
Oceania	87790.7 (68253.9 - 113046.1)	222966.5 (178508.8 - 281151.7)	153.98 (57.91 - 311.92)
South Asia	19066250.9 (15473211.0 - 23689539.5)	48132071.7 (39708182.2 - 59580155.0)	152.45 (67.62 - 285.05)
Southeast Asia	10619343.8 (8525534.4 - 13387139.9)	28443325.6 (22795528.1 - 35514785.4)	167.84 (70.28 - 316.57)
Southern Latin America	1619632.7 (1300608.0 - 2090006.1)	2964570.2 (2488244.4 - 3687630.6)	83.04 (19.05 - 183.53)
Southern Sub-Saharan Africa	686344.8 (533634.6 - 887490.8)	1516347.4 (1172984.5 - 1923538.5)	120.93 (32.17 - 260.46)
Tropical Latin America	3992553.0 (3184410.2 - 4980175.2)	12391214.1 (9934001.0 - 15189441.6)	210.36 (99.47 - 376.99)
Western Europe	16800967.1 (14054143.1 - 20311290.7)	24506384.5 (20991107.8 - 29418097.6)	45.86 (3.35 - 109.32)
Western Sub - Saharan Africa	2336793.9 (1858736.9 - 2949339.2)	5030556.8 (4041945.7 - 6346488.8)	115.28 (37.05 - 241.44)


**Table 2:** Incidence rates (%) of permanent tooth caries among adults aged ≥ 55 years and average annual percentage change (AAPC), 1990–2021

Characteristics	Incident Rate, % (95% Uncertainty Interval)		Average Apc, % (95% Ci)
	1990	2021	
**Overall**	18.1 (14.8 - 22.4)	17.5 (14.4 - 21.6)	-0.11 (-0.143 - -0.076)
**Sex**			
Female	18.0 (14.8 - 22.2)	17.5 (14.4 - 21.6)	-0.09 (-0.109 - -0.072)
Male	18.2 (14.8 - 22.8)	17.5 (14.3 - 21.6)	0.124 (-0.141 - -0.108)
**Age**			
55 - 59 years	21.1 (16.1 - 27.2)	21.0 (16.0 - 26.9)	-0.0136 (-0.0584 - 0.0311)
60 - 64 years	19.0 (14.6 - 24.3)	19.0 (14.7 - 24.3)	-7e-04 (-0.0329 - 0.0316)
65 - 69 years	17.3 (13.8 - 22.3)	16.8 (13.4 - 21.6)	-0.0966 (-0.1233 - -0.0699)
70 - 74 years	15.8 (12.1 - 21.0)	15.3 (11.9 - 20.1)	-0.1018 (-0.1448 - -0.0588)
75 - 79 years	15.3 (11.7 - 20.0)	14.2 (11.1 - 18.6)	-0.2417 (-0.2739 - -0.2095)
80 - 84 years	14.4 (11.0 - 18.5)	13.3 (10.2 - 17.1)	-0.2727 (-0.3045 - -0.241)
85 - 89 years	13.4 (10.3 - 17.2)	12.2 (9.5 - 15.5)	-0.3289 (-0.3688 - -0.289)
90 - 94 years	11.9 (8.5 - 15.7)	11.0 (7.9 - 14.5)	-0.2735 (-0.3252 - -0.2218)
95+ years	9.8 (5.4 - 15.1)	9.2 (5.2 - 14.1)	-0.2282 (-0.2785 - -0.1779)
**Sociodemographic Index Region**			
Low SDI	19.0 (15.6 - 23.2)	18.2 (15.0 - 22.3)	-0.133 (-0.155 - -0.111)
Low-to-middle SDI	20.5 (16.7 - 25.6)	20.2 (16.5 - 25.1)	-0.053 (-0.066 - -0.04)
Middle SDI	17.0 (13.7 - 21.3)	17.8 (14.3 - 22.3)	0.152 (0.112 - 0.191)
High SDI	17.4 (14.3 - 21.6)	16.2 (13.5 - 19.8)	-0.256 (-0.335 - -0.176)
High-middle SDI	18.4 (14.9 - 23.0)	16.3 (13.4 - 20.3)	-0.376 (-0.417 - -0.335)
**Global Burden of Diseases, Injuries, and Risk Factors Study Region**			
Andean Latin America	23.4 (18.4 - 29.5)	23.3 (18.2 - 29.2)	-0.016 (-0.029 - -0.002)
Australasia	18.3 (14.2 - 24.2)	17.9 (14.0 - 23.5)	-0.061 (-0.093 - -0.029)
Caribbean	27.3 (22.4 - 33.8)	25.5 (21.1 - 30.8)	-0.231 (-0.275 - -0.186)
Central Asia	24.3 (19.4 - 30.8)	24.5 (19.2 - 31.5)	0.01 (-0.014 - 0.033)
Central Europe	24.6 (20.1 - 30.5)	24.3 (20.1 - 30.2)	-0.026 (-0.066 - 0.015)
Central Latin America	20.8 (16.7 - 26.4)	20.8 (16.7 - 26.3)	0.005 (-0.009 - 0.018)
Central Sub-Saharan Africa	17.9 (13.8 - 23.7)	18.0 (14.0 - 24.1)	0.011 (-0.019 - 0.04)
East Asia	10.2 (8.0 - 13.0)	10.7 (8.4 - 13.7)	0.128 (0.017 - 0.24)
Eastern Europe	24.2 (19.3 - 31.2)	23.5 (18.9 - 29.8)	-0.091 (-0.122 - -0.06)
Eastern Sub-Saharan Africa	18.4 (15.0 - 22.5)	17.8 (14.7 - 22.0)	-0.105 (-0.146 - -0.064)
High-income Asia Pacific	17.4 (13.4 - 22.3)	14.1 (11.3 - 17.2)	-0.727 (-0.94 - -0.513)
High-income North America	17.3 (13.1 - 22.7)	17.4 (13.4 - 22.3)	-0.021 (-0.158 - 0.116)
North Africa and Middle East	22.2 (18.0 - 27.5)	22.0 (18.0 - 27.3)	-0.02 (-0.045 - 0.006)
Oceania	18.2 (14.2 - 23.5)	18.1 (14.5 - 22.8)	-0.012 (-0.059 - 0.034)
South Asia	20.1 (16.3 - 25.0)	19.4 (16.0 - 24.0)	-0.122 (-0.141 - -0.102)
Southeast Asia	25.1 (20.1 - 31.6)	24.8 (19.9 - 31.0)	-0.026 (-0.079 - 0.028)
Southern Latin America	20.4 (16.4 - 26.4)	20.1 (16.9 - 25.1)	-0.057 (-0.113 - -0.002)
Southern Sub-Saharan Africa	15.5 (12.1 - 20.1)	15.6 (12.0 - 19.8)	0.022 (-0.032 - 0.075)
Tropical Latin America	26.4 (21.0 - 32.9)	28.0 (22.4 - 34.3)	0.163 (0.061 - 0.264)
Western Europe	17.3 (14.5 - 20.9)	16.4 (14.1 - 19.7)	-0.172 (-0.2 - -0.144)
Western Sub - Saharan Africa	16.2 (12.9 - 20.4)	15.7 (12.6 - 19.7)	-0.107 (-0.151 - -0.063)


### Age-Specific Patterns

The 55–59 age group consistently maintained the highest caseload across the study period, rising from 39.13 million cases (95% UI: 29.84 to 50.30) in 1990 to 83.07 million cases (95% UI: 63.17 to 106.52) in 2021, a 112.4% increase (see Table 1). While all age groups demonstrated case number growth, the most dramatic increase occurred in the ≥ 95 years cohort (100067.2 cases in 1990 vs. 502211.4 in 2021). Nevertheless, this oldest demographic still exhibited substantially lower absolute case numbers compared to younger elderly populations (see Table 1). Contrasting with case count patterns, age-specific incidence rates displayed universal declines. Notably, the 80–84 age group experienced the most pronounced reduction in incidence rates (–0.27%) during the observation period (see Table 2).

### Sex Differences

Gender differences in incidence rates were significant, with the APC of female incidence rates continuously decreasing at –0.27% (95% CI –0.33% to –0.22%) from 2017 to 2021, while male incidence rates counter-cyclically increased at APC of 0.32% (95% CI 0.09% to 0.54%) from 2019 to 2021 (see Table A1 and Figs 1 to 3).

### Regional Trends

**Fig 4:**
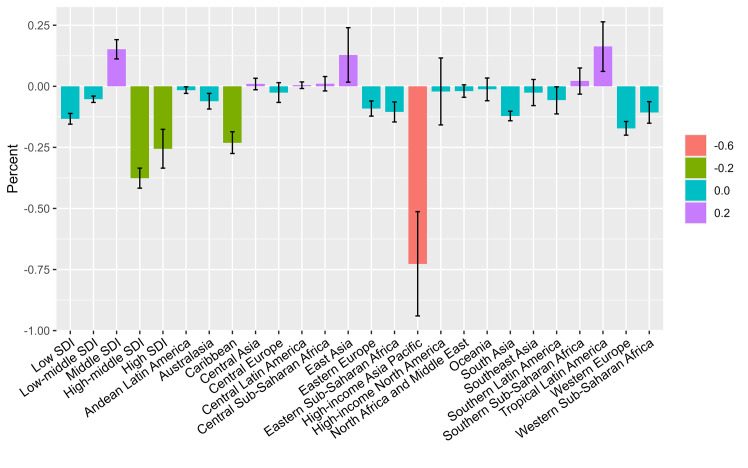
Average annual percentage change in global incidence rate of caries in permanent teeth in people aged over 55 years by different regions and SDIs from 1990 to 2021. SDI: Sociodemographic index.

In 1990, the incidence of permanent tooth caries in older people over 55 years of age ranged from 17.0% (95% CI 14.3% to 21.6%) in a high SDI area to 20.5% (95% CI 16.7% to 25.6%) in a low-to-middle SDI area (see Table 2). In 2021, the incidence ranged from 16.2% (95% CI 13.5% to 19.8%) in high SDI areas to 20.2% (95% CI 16.5% to 25.1%) in low-to-middle SDI areas (see Table 2). Although incidence rates declined in most SDI categories over the study period, the number of permanent tooth caries increased significantly in the aged group over 55 years old, with the largest increase in cases in middle SDI areas (+183.71%) and the smallest increase in high SDI areas (+72.41%) (see Table 1 and Fig 4).

Notably, since 1990–2021, the trend of population ageing has become increasingly evident. The incidence of permanent tooth caries in older people in low-to-middle SDI areas was consistently significantly higher than in other areas. Trend analysis showed decreasing incidence in low SDI (AAPC –0.13%, 95% CI –0.16% to –0.11%), low-to-middle SDI (AAPC –0.05%, 95% CI –0.07% to –0.04%) and high SDI regions (AAPC –0.26%, 95% CI –0.34% to –0.18%). In contrast, the middle SDI region demonstrated an increasing trend (AAPC 0.152%, 95% CI 0.11% to 0.19%) (see Table 2).

In recent years, the incidence rates of dental caries in elderly groups in high SDI areas increased by 0.69% (95% CI 0.4% to 0.99%), whereas low, middle, and high-to-middle SDI regions showed declines of 0.14% (95% CI –0.19% to –0.08%), 0.35% (95% CI –0.42% to –0.29%) and 0.40% (95% CI –0.59% to –0.2%), respectively (see Table A1 and Fig 4).

At the GBD regional level, the number of incidence cases of those aged 55 years and older increased in all 21 regions from 1990 to 2021 (see Table 1). Meanwhile, with the largest increase (AAPC 0.163%, 95% CI 0.06% to 0.26%), the incidence of permanent dental caries in Tropical Latin America is far ahead, rising from 26.6% (95% CI 21.0% to 32.9%) in 1990 to 28.0%(95% CI 22.4% to 34.3%) in 2021. followed by Andean Latin America (0.128%) (95% CI 0.06% to 0.26%). The incidence rate in East Asia also increased, with an AAPC of 0.13% (95% CI 0.02% to 0.24%). In contrast, East Asia has the lowest overall incidence rates, with 10.2%(95% CI 8.0% to 13.0%) in 1990 and 10.7% (95% CI 8.4% to 13.7%) in 2021. Two regions, Tropical Latin America and East Asia, demonstrated sustained upward trends over the study period. Conversely, 10 regions exhibited decreasing trends, prominently featuring High-Income Asia Pacific (AAPC –0.73%, 95% CI –0.94% to –0.51%) and the Caribbean (AAPC –0.23%, 95% CI –0.28% to –0.17%) as the steepest reductions (Fig 4). Nine GBD regions, including Central Latin America and Central Sub-Saharan Africa, maintained minimal changing incidence rates throughout the observation period (see Table 2).

Temporal analysis revealed evolving patterns: recent data showed stabilisation in 9 regions (eg, South and Central Asia), declines in 10 regions, and increases in 2 regions (see Table 2).

### National Trends

In 2021, China (41.01 million cases) and India (39.75 million cases) accounted for approximately one-third of the global number of dental caries cases among individuals aged 55 and older (121.54 million cases) (see Table 1 and Table A2). Compared with 1990, the number of cases increased in 202 countries in 2021, with the largest increases in Qatar (872.01%), the Kingdom of Bahrain (479.825%) and Kuwait (410.29%), and only 1 country showed a decrease in the number of cases, which was Georgia (–6.67%) (see Table 1 and Fig 2).

The above-mentioned growth trends may be related to the increase in the total global population and the aggravation of population ageing.

In 2021, the incidence rates of dental caries in people over 55 years of age in various countries or regions were between 13.7% and 29.9%. There were three countries or regions with the highest incidence, including Saudi Arabia (29.9%), Saint Vincent and the Grenadines (28.3%) and Belize (28.3%). The incidence rates in countries in West Asia, the Middle East, the Mediterranean coast, Eastern Europe, Africa, and Japan also remain at a relatively high level (see Fig 1 and Table A3). This might be related to factors such as local dietary habits or limited medical conditions. Epidemiological surveillance data (1990–2021) reveal distinct geographic disparities in global dental caries prevalence. Forty-two countries/regions exhibited upward trajectories, with Sweden demonstrating the most pronounced annualised rate increase (0.365%). Conversely, Trinidad and Tobago recorded the steepest decline among 69 decreasing regions, showing a substantial average annual reduction of 1.904% (see Table A3).

Focusing on recent epidemiological trends among adults aged ≥ 55 years, 42 countries/regions displayed ascending incidence patterns. Belgium (3.13%) and South Korea (2.5%) emerged as the fastest-growing regions. Parallel analysis identified 69 countries/regions with declining rates, featuring Syria (–1.71%) and Trinidad and Tobago (–1.82%) as the most significant reducers–the latter maintaining consistent downward trends across multiple observation periods. Notably, 91 countries/regions maintained stable incidence thresholds during this period, including Belgium (–0.096%) and Vietnam (–0.014%) (see Table A3).

## DISCUSSION

This study provides a comprehensive assessment of long-term trends in the incidence of permanent tooth caries among adults aged ≥ 55 years from 1990 to 2021 using GBD 2021 data. The absolute number of incident cases increased substantially over the study period (from 121.54 million to 260.18 million in 2021), with marked heterogeneity across sociodemographic regions and age strata. These findings highlight the dual forces shaping oral disease burden in older adults: demographic expansion and epidemiological transition.

The increase in incident case numbers is largely attributable to global population ageing and demographic expansion. Improvements in life expectancy and greater retention of natural teeth into older age mean that more individuals remain at risk of developing caries later in life.^8,24,30^ While tooth loss historically reduced caries risk in advanced age groups, contemporary cohorts retain more teeth^8,24,30^ and are therefore exposed to cumulative caries risk over a longer lifespan.^24,30^ This epidemiological shift underscores the growing importance of caries prevention in geriatric populations.

The marked regional disparities observed in this study likely reflect complex interactions between preventive coverage,^12,18,19,22,26^ fluoride exposure,^19,21^ dietary patterns,^9,18,22,23,29^ socioeconomic development,^12,25^ and access to dental care services.^12,18,19,22,25,26^ In high SDI regions, the generally stable or declining trends may be attributed to sustained public health investment in preventive dentistry over several decades. Widespread use of fluoridated toothpaste, implementation of community water fluoridation programmes in some countries, school-based preventive initiatives, and regular access to restorative care have collectively contributed to improved caries control.^20,33^ Furthermore, higher levels of oral health literacy and routine dental attendance among older adults in these settings may facilitate earlier detection and management of lesions, preventing progression to more severe disease.

Middle and low SDI regions consistently exhibited higher incidence rates than other regions. These patterns may be associated with rapid socioeconomic transition and urbanisation, often accompanied by increased consumption of refined carbohydrates and free sugars. Nutrition transition, particularly the growing availability of processed foods and sugar-sweetened beverages, has been consistently linked to rising caries risk.^9,18,22,23,29^ At the same time, preventive infrastructure in many of these regions remains underdeveloped. Limited access to fluoridated products,^19,21^ lower availability of professional preventive services,^17,19^ financial barriers to dental care,^12,25^ and workforce shortages^19^ may all contribute to insufficient disease control.

Importantly, improvements in tooth retention in older populations may paradoxically increase the pool of teeth at risk for caries in settings where preventive systems have not developed at a comparable pace. As more adults retain their natural dentition into advanced age, root surface exposure due to gingival recession, xerostomia related to polypharmacy, and systemic comorbidities may further elevate susceptibility to caries.^5^ Without adequate preventive support, these biological vulnerabilities may amplify the impact of environmental and behavioural risk factors.^5,19^

Taken together, these findings align with previous epidemiological studies suggesting that caries trends are strongly shaped by social determinants of health and the organisation of oral healthcare systems. The persistence of widening inequalities underscores the need for context-specific preventive strategies that address both behavioural risk factors and structural barriers to care.

Joinpoint analysis revealed distinct temporal change points in multiple regions, suggesting that epidemiological trajectories are not linear and may correspond to policy changes, economic transitions, or shifts in population health behaviours. These findings highlight the importance of continuous surveillance and region-specific strategies rather than assuming uniform global trends.

From a preventive dentistry perspective, the findings emphasise the need to strengthen oral health promotion and caries prevention strategies targeting older adults. Preventive measures should include improved fluoride exposure, early detection of root caries, dietary counselling, management of xerostomia, and integration of oral health services within general geriatric care frameworks.^1,14,16,20,33^ Community-based programmes and primary care integration may be particularly important in settings where access to specialist dental services is limited.^1,14,16^

Several limitations should be acknowledged. First, the epidemiological estimates were derived from the Global Burden of Disease (GBD) modelling framework rather than direct clinical measurements, which may introduce uncertainty. At the same time, many methodological adjustments, including misclassification correction and reallocation, etc., were applied to enhance consistency with empirical surveillance data. Previous validation studies have demonstrated satisfactory concordance between GBD projections and regional registries.^10,25^ A second analytical limitation stems from the absence of dentition-specific caries localisation data within the GBD databases. Consequently, our analytical model cannot elucidate potential variations in caries progression rates associated with distinct morphological characteristics of molars, premolars, or anterior teeth.

In conclusion, the global burden of permanent tooth caries among adults aged ≥55 years remains substantial and demonstrates significant regional variation. As populations continue to age and retain more natural teeth, targeted and context-specific preventive strategies will be essential to reduce inequalities and promote healthy ageing in oral health.

## CONCLUSION

The global burden of permanent tooth caries among adults aged ≥55 years remains substantial and demonstrates pronounced regional and sociodemographic disparities. Although age-standardised incidence rates were stable or declining in some high SDI regions, increasing trends in several low and middle SDI settings highlight persistent inequalities in preventive coverage and access to care.

As populations continue to age and retain more natural teeth, the demand for effective caries prevention strategies in older adults will increase. Strengthening fluoride-based prevention, early detection of root caries, and integration of oral health into geriatric healthcare services are essential to promote healthy ageing and reduce global disparities in oral health.^12,18,19,22,26^

### Acknowledgements

We would like to thank the staff of the Department of Stomatology at Nanfang Hospital who helped make this study possible.

#### Funding statement

This work was supported by the National Natural Science Foundation of China (82100997), the Clinical Research Program of Nanfang Hospital, Southern Medical University (2021CR011), the College Students’ Innovative Entrepreneurial Training Plan Program (202412121284, S202312121173, 202212121240), the Science and Technology Program of Maoming (2022S020) and the Project of Teaching Quality and Teaching Reform, Southern Medical University (ZL2025171).

#### Competing interests

The authors declare that they have no known competing financial interests or personal relationships that could have appeared to influence the work reported in this paper.

#### Patient consent for publication

Not applicable.

#### Ethics approval

The study used publicly available secondary data and did not require ethical approval.

#### Data sharing statement

Data are available in a public, open-access repository. Data are available in a public, open-access repository. The data that support the findings of this study are accessible online at https://vizhub.healthdata.org/gbd-results/. This public link to the database of the GBD study is open, and the use of data does not require additional consent from IHME.

### Appendix

https://www.quintessence-publishing.com/quintessenz/journals/articles/downloads/ohpd_2026_8393_sun_table_a1.pdf

https://www.quintessence-publishing.com/quintessenz/journals/articles/downloads/ohpd_2026_8393_sun_table_a2.pdf

https://www.quintessence-publishing.com/quintessenz/journals/articles/downloads/ohpd_2026_8393_sun_table_a3.pdf

https://www.quintessence-publishing.com/quintessenz/journals/articles/downloads/ohpd_2026_8393_sun_table_a4.pdf
